# DNA Repair Syndromes and Cancer: Insights Into Genetics and Phenotype Patterns

**DOI:** 10.3389/fped.2020.570084

**Published:** 2020-10-23

**Authors:** Richa Sharma, Sara Lewis, Marcin W. Wlodarski

**Affiliations:** ^1^Department of Hematology, St. Jude Children's Research Hospital, Memphis, TN, United States; ^2^Department of Oncology, St. Jude Children's Research Hospital, Memphis, TN, United States; ^3^Division of Pediatric Hematology and Oncology, Department of Pediatrics and Adolescent Medicine, Medical Center, Faculty of Medicine, University of Freiburg, Freiburg, Germany

**Keywords:** DNA repair, cancer predisposition, hematological malignances, hereditary cancer, pediatric cancer

## Abstract

DNA damage response is essential to human physiology. A broad spectrum of pathologies are displayed by individuals carrying monoallelic or biallelic loss-of-function mutations in DNA damage repair genes. DNA repair syndromes with biallelic disturbance of essential DNA damage response pathways manifest early in life with multi-systemic involvement and a high propensity for hematologic and solid cancers, as well as bone marrow failure. In this review, we describe classic biallelic DNA repair cancer syndromes arising from faulty single- and double-strand DNA break repair, as well as dysfunctional DNA helicases. These clinical entities include xeroderma pigmentosum, constitutional mismatch repair deficiency, ataxia telangiectasia, Nijmegen breakage syndrome, deficiencies of DNA ligase IV, NHEJ/Cernunnos, and ERCC6L2, as well as Bloom, Werner, and Rothmund-Thompson syndromes. To give an in-depth understanding of these disorders, we provide historical overview and discuss the interplay between complex biology and heterogeneous clinical manifestations.

## Introduction

Preservation of genomic DNA is fundamental to maintenance of life. Mammalian DNA can withstand at least 10^5^ lesions in a single cell per day caused by intrinsic biological processes and extrinsic genotoxic agents (1). DNA repair mechanisms are highly complex and conserved pathways that have evolved over time. Their role is to restore genomic damage so that naturally occurring DNA lesions are rapidly neutralized and transmission of accurate genetic code across generations can occur (2). In this review, we discuss biological and clinical features of classic DNA repair disorders that predispose to hematologic and solid cancers early in life. Due to intricate genetic underpinnings and heterogeneous clinical manifestations, the diagnosis of these underappreciated syndromes is challenging and typically requires a high index of suspicion. Insight into specific phenotype spectrum and associated cancers can increase awareness of these rare syndromes. As a result, a timely diagnosis and multidisciplinary management with focus on structured surveillance can improve life expectancy in this pediatric population.

**Figure 1:**
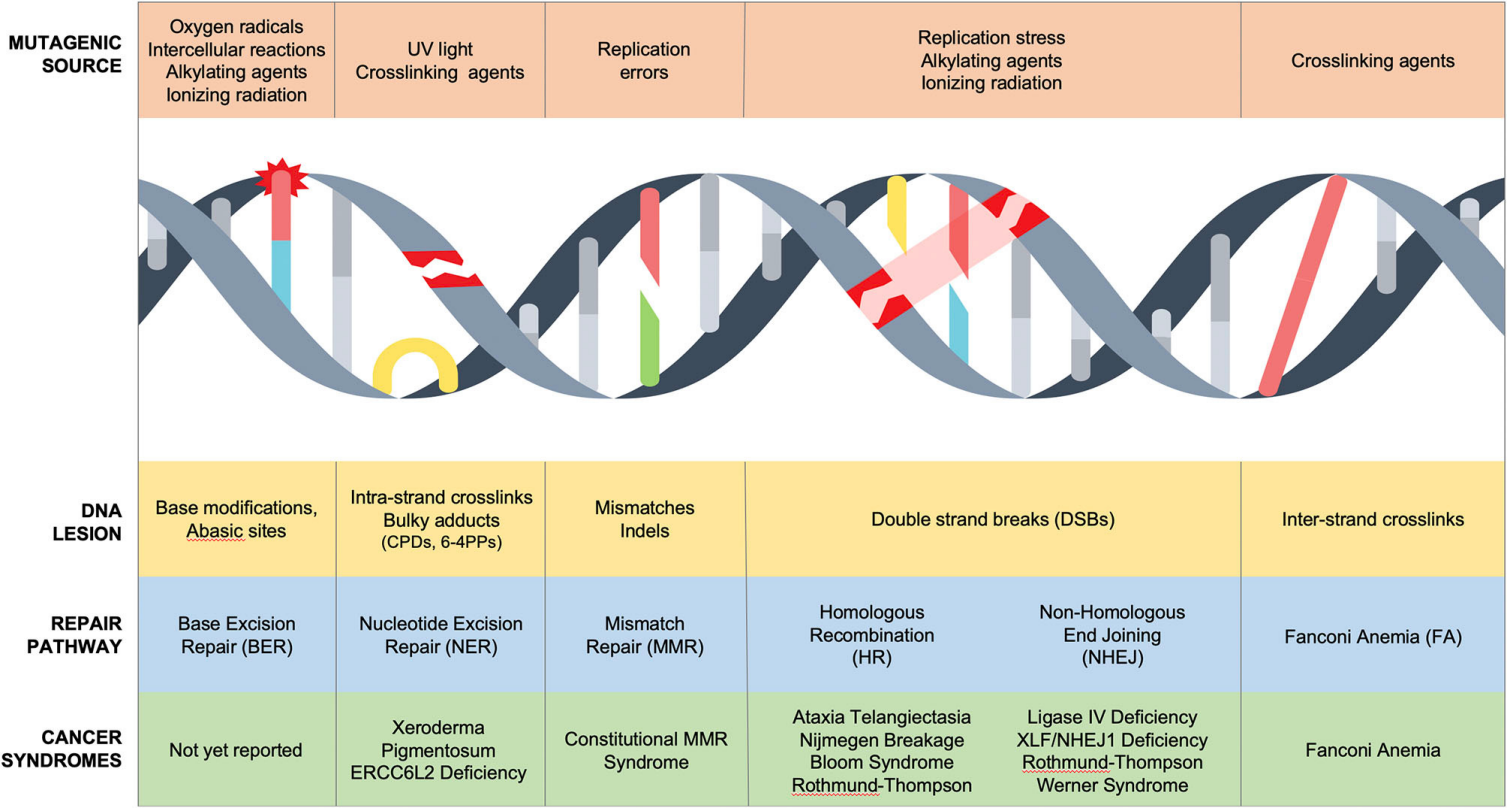
DNA repair disorders associated with cancer predisposition in pediatric population. Several DNA damage sources cause unique DNA lesions that are repaired by specific DNA repair pathways. Biallelic mutations in NER, MMR, HR, NHEJ, and FA/HR cause cancer predisposition syndromes of childhood.

Sources of DNA damage are constant, innumerable, and divided into endogenous and exogenous culprits. Endogenous damage is caused by replication errors, as well as reactive intermediates secondary to essential cellular chemical reactions (reactive oxygen species, aldehydes). Exogenous damaging agents include ultraviolet (UV) and ionizing radiation, environmental chemicals (polycyclic aromatic hydrocarbons, benzo[a]pyrene, aromatic compounds), and chemotherapeutic agents including DNA-alkylators (temozolomide), DNA crosslinkers (mitomycin C or cisplatin), topoisomerase inhibitors (etoposide), and radiomimetics (bleomycin) (2–4). These often unavoidable insults cause toxic DNA intermediates such as single-nucleotide lesions, helical distorting adducts and dimers, single-strand breaks (SSBs), and double-stranded breaks (DSBs), all of which activate the DNA damage response (Figure 1) (5).

The DNA damage response is a molecular surveillance system that regulates cell cycle progression at G1-S, intra-S, and G2-M checkpoints to maintain genomic stability (6). Heritable genetic mutations in this safeguard infrastructure results in cancer predisposition syndromes (5). Li-Fraumeni syndrome (LFS) is the prototypical cancer susceptibility disorder characterized by early onset of solid and hematological cancers due to germline monoallelic mutations in p53, a tumor suppressor gene (7) [excellent reviews can be found elsewhere (8)]. LFS highlights the central role of p53 as a bona fide genome guardian, which modulates G1-S and G2-M checkpoints in response to DNA damage pathways (9, 10). At least eight DNA repair mechanisms have been described to orchestrate the repair of mammalian DNA in a cooperative and redundant fashion (2). Importantly, nucleotide excision repair (NER), mismatch repair (MMR), homologous recombination (HR), non-homologous end joining (NHEJ), and inter-strand DNA crosslink repair have been associated with Mendelian syndromes with cancer predisposition in children (Figure 1, Table 1).

**Table 1:** DNA repair deficiencies in single strand and double strand DNA repair and RECQ helicases result in classic DNA repair syndromes with multisystemic manifestations and oncogenic predisposition.

**DNA repair pathway**	**Associated syndrome**	**Expected biallelic mutations**	**Clinical testing**	**Clinical features**	**Malignancy spectrum**
**SINGLE STRAND BREAK REPAIR DISORDERS**					
NER^#^	Xeroderma Pigmentosum	*XPA, XPB, XPC, XPD, XPE, XPF, XPG, XPV*	Screening: UV hypersensitivity Confirmation: genetic testing	Skin Ocular Neurologic	Major: SCC, BCC, melanoma Minor: AML/MDS, brain/spinal cord
	ERCC6L2 deficiency	*ERCC6L2*	Genetic testing	Neurologic Bone marrow failure	MDS, erythroleukemia
MMR	Constitutional mismatch repair disorder	*MLH1, MSH2, MSH6, PMS2*	Screening: IHC, MSI, hypermutation (>100/MB) Confirmation: genetic testing	Skin	Major: brain, GI, T-NHL, ALL, AML Minor: sarcomas, GU
**DOUBLE STRAND BREAK REPAIR DISORDERS**					
HR	Ataxia telangiectasia	*ATM*	Screening: TREC, AFP, telomere length, t(7;14) Confirmation: Genetic testing	Neurologic Immunologic Endocrine	Major: B-NHL, HL, ALL, breast Minor: gastric, brain
	Nijmegen breakage syndrome	*NBN*	Screening: TREC, AFP, telomere length, t(7;14) Confirmation: Genetic testing	Neurologic Endocrine Immunologic	Major: B-NHL, T-LBL Minor: HL, ALL, AML, brain tumors, sarcoma
NHEJ	DNA Ligase IV Deficiency syndrome	*LIG4*	Screening: TREC Confirmation: Genetic testing	Endocrine Immunologic Bone marrow failure	Major: ALL, B-NHL Minor: AML, MDS
FA	Fanconi anemia	22 FA genes^*^	Screening: Chromosomal breakage, AFP, telomere length Confirmation: Genetic testing	Congenital anomalies Bone marrow failure Endocrine	Major: SCC (head/neck), AML, MDS Minor: anogenital
**RECQ HELICASE DEFICIENT REPAIR DISORDERS**					
HR	Bloom syndrome	*BLM*	Screening: SCEs, telomere length Confirmation: Genetic testing	Endocrine Skin Immunologic	Major: AML, ALL, B-NHL, colorectal Minor: breast, SCC, BCC, Wilm's
HR, NHEJ	Werner syndrome	*WRN*	Screening: telomere length Confirmation: Genetic testing	Aging, premature Heart Endocrine	Major: thyroid follicular carcinoma Minor: melanoma, sarcomas, MDS, AML
	Rothmund-thompson syndrome	*RECQL4*	Confirmation: Genetic testing	Skin Ocular	Major: Osteosarcoma, BCC, SCC, melanoma Minor: AML, MDS, lymphoma^**^
	Rapadilino			Endocrine Skeletal anomalies	Major: lymphoma^**^, osteosarcoma
	Baller-gerold syndrome			Skeletal anomalies	NK/T cell lymphoma

# Cockayne syndrome and Trichothiodystrophy are important NER deficient syndromes that do not exhibit cancer predisposition risk.

* Includes following 22 genes: FANCA, FANCB, FANCC, FANCD1 (BRCA2), FANCD2, FANCE, FANCF, FANCG, FANCI, FANCJ (BRIP1), FANCL, FANCM, FANCN (PALB2), FANCO (RAD51C), FANCP (SLX4), FANCQ (ERCC4), FANCR (RAD51), FANCS (BRCA1), FANCT (UBE2T), FANCU (XRCC2), FANCV (REV7).

** = types of lymphomas not reported in literature.

*NER, nucleotide excision repair; MMR, mismatch repair; HR, homologous recombination; NHEJ, non-homologous end joining; FA, Fanconi anemia; RAPADILINO, (**RA**dial **RA**y defect; **PA**tellae hypoplasia or aplasia and cleft or highly arched **PA**late; **DI**arrhea and **DI**slocated joints; **LI**ttle size and **LI**mb malformation; **NO**se slender and **NO**rmal intelligence) syndrome; XPA-G, xeroderma pigmentosum A-G; XPV, xeroderma pigmentosum V; MLH1, MutL homolog 1; MSH2, MutS homolog. 2; MSH6, MutS homolog 6; PMS2, PMS1 homolog 2; ATM, Ataxia telangiectasia mutated; NBN, Nibrin; LIG4, DNA ligase 4; BLM, Bloom syndrome RecQ like helicase; WRN, Werner syndrome RecQ like helicase; RECQL4, REQ like helicase 4; UV, ultra-violet; IHC, immunohistochemistry; MSI, microsatellite instability; TREC, T cell receptor excision circles; AFP, Alpha-fetoprotein; SCEs, sister chromatid exchanges; SCC, squamous cell carcinoma; BCC, basal cell carcinoma; GI, gastrointestinal; GU, genitourinary; ALL, acute lymphoblastic leukemia; AML, acute myeloid leukemia; MDS, myelodysplastic syndrome; NHL, non-Hodgkin lymphoma; HL, Hodgkin lymphoma; LBL, lymphoblastic lymphoma*.

Although classic DNA repair syndromes affect pediatric population, their rarity, complex genetics, and heterogeneous phenotypic features make them underrecognized. In the following, we highlight other (non-FA) DNA repair pathway deficiencies and the resulting clinical manifestations in hopes of minimizing missed opportunities for early diagnosis and risk-adapted treatment of aggressive cancers that increase morbidity and mortality in this biologically distinct patient population.

## Syndromes Caused by Faulty Single Strand Break Repair

SSBs are the most common type of DNA lesion that represent discontinuity in one of the two strands of the DNA helix (11). Single-strand lesions induce replication block and can progress to lethal DSBs if unrepaired in active replicating cells (12) while causing cell death in post-mitotic cells (13, 14). Three repair mechanisms, BER, MMR, and NER, have evolved to mitigate single-strand breaks. BER ameliorates single base damage [detailed review available (15)], which when abrogated can lead to colorectal cancers in adults (16, 17) without evidence to cause childhood cancers. In contrast, both MMR, which resolves base mismatch and insertions–deletions (indels), and NER, which resolves bulky helix distorting lesions, are associated with pediatric cancer predisposition syndromes (Figure 1).

### Xeroderma Pigmentosum (XP)

XP, the first DNA repair disorder described in 1874 by Hebra and Kaposi (18), is an autosomal recessive syndrome with dermatological, ocular, and neurological manifestations with skin cancer predisposition (Table 1). XP is estimated to affect 1 per million in the United States and 2.3 cases per million in Western Europe (19, 20) with higher prevalence in Japan (21) and North Africa (22). XP patients are unable to repair UV radiation-induced DNA damage due to mutations in the NER pathway. Biallelic mutations in one of the eight XP genes [*XPA*-*G* and *XP-variant(V*)] of the NER pathway cause classic XP (23). Mutations in *XPA* through *XPG* account for about 80% of XP cases with the remaining attributed to *XPV* (24). Patients commonly present by 2 years of age with increased number of lentigines (freckle-like pigmentation) in sun-exposed areas, a diagnostic skin finding in XP. Extreme sensitivity to sunlight resulting in acute severe sunburns is the presenting feature in 50% of patients. Increased sun exposure and lack of sun protection correlates with development of telangiectasias, pigmented seborrheic warty lesions, and atrophic skin (20, 25). Patients with mutations in *XPA, XPB, XPD, XPF*, and *XPG* have severe photosensitivity at a young age (26). Photophobia is often present with ocular abnormalities limited to UV-exposed areas including eyelids, cornea, and conjunctiva (27). *XPC* patients are specifically hypersensitive to ocular damage with severe keratitis, corneal opacification, and vascularization (24). Approximately one third of patients exhibit progressive neuronal degeneration with *XPA, D*, and *G* groups considered to be the most severely affected (28). Clinical presentations can be as subtle as loss of deep tendon reflexes and high-frequency sensorineural hearing to intellectual disability, motor dysfunction (spasticity, ataxia, difficulties swallowing), and frank quadriparesis (25, 26, 29, 30).

XP patients have an estimated 10,000-fold greater risk of developing basal cell and invasive squamous cell carcinomas compared to the general population, with median onset age of <10 years (29). The risk of melanoma has been estimated to be 2,000-fold higher, with median age of onset of 20 years (29). Interestingly, *XPC, XPE*, and *XPV* mutations, which are classified as mild XP group due to only minor photosensitivity without neurological abnormalities, show the highest penetrance for cancers (24, 28). This is thought to be due to rapid accumulation of UV damage without sun protection in this patient population who lack overt skin findings resulting in late diagnosis (24). Mucosal cancers of the tongue, myelodysplastic syndrome (MDS), acute myeloid leukemia (AML), and tumors of the brain and spinal cord have also been described in XP patients (20, 24, 29, 31–33). Importantly, *TP53* somatic alterations are exceptionally common in XP-associated skin tumors and MDS/AML with high rate of del5q and del7q karyotype alterations in XP-C patients (33, 34). The broad phenotype spectrum seen in XP is a direct consequence of NER deficits at the molecular level. The NER pathway is orchestrated by 30 proteins, and two subbranches, namely, global genomic repair and transcription-coupled repair, recognize and remove UV-induced cyclobutene pyrimidine dimers (CPD) and 6-4 pyrimidine-primidone (6-4PPs) dimers. Global genomic repair relies on XPC and XPE to sense DNA adducts while transcription-coupled repair recognizes damage on the transcribed strand using NER proteins: Cockayne syndrome A and B (CSA, CSB). Both sub-pathways converge to recruit XPD and XPB helicase-containing transcription complex to unwind damaged DNA. This allows XPA to secure single-strand DNA followed by incision of damaged DNA portion by endonucleases XPF/ERCC1 and XPG and gap filling by replication polymerases (35, 36). XPV/POLH is involved in replicating past unrepaired UV-induced thymine dimers or AP sites during translesion synthesis (37, 38). Of note, Cockayne syndrome (39) and Trichothiodystrophy (40) are important NER-deficient syndromes that do not exhibit cancer predisposition risk.

### ERCC Excision Repair 6 Like 2 (ERCC6L2) Deficiency

Biallelic loss-of-function mutations in ERCC6 like 2 (*ERCC6L2*) have been associated with BMF, MDS, and acute erythroid leukemia (AML M6). ERCC6L2 is a Snf2 helicase that belongs to SWI/SNF protein family, which makes chromatin accessible to transcription machinery (41). Along with its role in RNA processing, ERCC6L2 plays a role in DNA repair by facilitating cross talk between transcription-coupled NER and NHEJ DNA repair pathways. Specifically, ERCC6L2 repairs transcription-affiliated DNA lesions through its interaction with DNA-PK (42), a central component of the NHEJ DNA repair complex (43). The first report linked homozygous truncating *ERCC6L2* mutations to a bone marrow failure (BMF) syndrome manifesting with neurological and developmental findings in three index cases (9, 12, and 19 years of age) from consanguineous families (44). In another study, 7 patients, with median age of 13 years, were described to have hypocellular marrow in the setting of biallelic *ERCC6L2* mutations, 2 of which displayed dysplastic marrow features with monosomy 7 (45). Of note, only 1 patient from a consanguineous family had neurological and developmental delays. Most recently, biallelic germline mutations were identified in five patients with the unique phenotype of acute erythroleukemia with median age of onset at 49 years. Additionally, all *ERCC6L2*-mutated acute erythroleukemia cases harbored somatic *TP53* mutations at diagnosis (46). It remains to be answered if ERCC6L2 also plays a role in solid tumor predisposition and other types of hematologic malignancies.

### Constitutional Mismatch Repair Deficiency (CMMRD)

CMMRD is a recessively inherited, cancer predisposition syndrome, which was described initially in 1999 (47, 48) and affects 1 in 1 million children (49). CMMRD is characterized by childhood onset of broad-spectrum malignancies secondary to biallelic (homozygous or compound heterozygous) germline mutations in the MMR pathway genes, mutL homolog 1 (*MLH1*), mutS homolog 2 (*MSH2*), mutS homolog 6 (*MSH6*), and PMS1 homology 2 (*PMS2*) (50, 51). Parental consanguinity enriching for a founder mutation is observed in over 50% of CMMRD cancers (52, 53). However, in Western countries, genotypes with compound heterozygous mutations among non-consanguineous families are more common (54). In adults, monoallelic (heterozygous) mutations in these MMR genes are known to cause Lynch syndrome (LS), with predisposition primarily to colorectal, and endometrial cancers (55, 56).

The biological relevance of the MMR pathway is underscored in CMMRD patient tumors, which have a hypermutator phenotype (defined as >10 mutations/Mb), as a result of the inability for MMR machinery to identify and excise DNA damage. Specifically, MSH2–MSH6 heterodimer recognizes base–base mismatch and MSH2–MSH3 heterodimer detects large indel mismatch followed by mismatch excision by MLH1–PMS2 (50). Abrogation of the essential MMR genes leaves behind a trail of incorrect base incorporation and indels, especially in microsatellite regions resulting in increased mutational burden and microsatellite instability, diagnostic hallmarks of CMMRD tumors. Finally, gap filling is accomplished by DNA polymerases *epsilon* (POLE) and *delta* (POLD1), which can acquire somatic mutations during tumorigenesis resulting in “ultra-hypermutated” (>100 mutations/Mb) CMMRD tumors (57, 58). POLE/POLD1 deficiency has been considered as a cancer susceptibility syndrome since mutation carriers with colonic and extra-colonic tumors have been reported (59–62). Importantly, childhood colorectal carcinoma and medulloblastoma in the setting of biallelic POLE mutations have been described (63, 64). Of note, heterozygous germline deletion of *EPCAM*, which causes epigenetic silencing of MSH2, thereby conferring an increased risk of colorectal cancer (65), in addition to biallelic mutation of MSH3, resulting in colorectal cancer (66), has expanded the spectrum of MMR deficient malignancies in humans.

CMMRD patients develop devastating malignancies at an early age with a median onset of 7.5 years (53). The cancer spectrum includes CNS tumors (estimated prevalence of 50%), digestive tract tumors (40%), hematological malignancies (33%), and other solid cancers (67). In a cohort study with 31 patients, the median age at diagnosis of hematologic malignancies, brain tumors, and gastrointestinal cancers was 6.6, 10.3, and 16 years, respectively (54). Commonly encountered brain tumors are high-grade gliomas with few reports of low-grade gliomas, CNS embryonal tumors, and medulloblastoma (49, 68, 69). Prevalent hematological malignancies are non-Hodgkin lymphoma (NHL), particularly T-lymphoblastic NHL followed by T cell-acute lymphoblastic leukemia (T-ALL) and AML (49, 53, 70). The affected MMR gene correlates with the cancer spectrum. MSH6 and/or PMS2 biallelic mutations “favor” brain tumors while MLH1 or MSH2 mutations are biased for development of aggressive hematological malignancies (53, 68). Greater than 40% of PMS2-mutated patients develop secondary neoplasms. However, MLH1/MSH2 patients have a secondary malignancy risk of 22% due to poor survival from the first malignancy (53, 68). Expectedly, colorectal carcinoma, the most prevalent Lynch syndrome associated cancer, has higher prevalence in CMMRD patients with biallelic MSH6 or PMS2 mutations (49, 53). Other solid tumors include osteosarcoma, rhabdomyosarcoma, neuroblastoma, and Wilms tumor (53).

Outside of cancers, certain features are recurrently found in patients with CMMRD. Many patients present with dermatological manifestations such as café-au-lait macules (CALMs), hyper- and hypopigmented skin alterations, venous anomalies, and pilomatricomas (benign hair follicle tumor). At least one CALM or hyperpigmented skin area is found in more than 60% of patients (53). Agenesis of the corpus callosum and mild immunodeficiency with decreased levels of immunoglobulins IgG and IgA were previously described (53). Collectively, oncologic and non-oncologic clinical criteria are used in a three-point scoring system established by the European consortium “Care for CMMRD” (C4CMMRD) for diagnosis of CMMRD (53).

## Syndromes Caused by Faulty Double-Stranded Break Repair

DSBs are the most destructive DNA lesions, which, when left unattended, result in cell death. HR and NHEJ are the two main DSB DNA repair pathways that differ in key aspects. HR is a high-fidelity repair pathway that dominates during S and G2 phase to repair DSB damage and relies on the presence of sister chromatids (71). In addition, it regulates essential cellular processes like meiotic recombination (72). On the other hand, an error-prone NHEJ pathway is active throughout the cell cycle (dominating in G1) and directly ligates two broken ends of a DSB. Outside of DNA repair, it is involved in T-cell receptor and immunoglobulin repertoire generation (73). The ability to resolve high-stake DSBs in a time-sensitive manner makes NHEJ a ubiquitous DSB repair pathway (74).

Since its first description by the Swiss pediatrician Guido Fanconi (75), Fanconi Anemia (FA) has been used as the prototypical example of a DSB repair syndrome associated with cancer. FA pathway recognizes and repairs toxic DNA inter-strand crosslinks that induce a replication block followed by formation and repair of DSBs. The inability to resolve these crosslinks results in FA, a cancer predisposition syndrome caused by biallelic mutations in 1 of 22 FA genes (76–81). FA usually manifests early in life with congenital anomalies involving many organ systems, progressive BMF and a very high risk for the development of MDS, AML, head and neck carcinomas, as well as multiple other cancer types. A number of comprehensive studies and reviews on FA and FA-associated cancers have been published elsewhere (82–85).

We will review defects in the DNA repair machinery proteins of the HR system (ATM, NBN) and the NHEJ pathway (LIG4, NHEJ1, Artemis) that result in rare cancer predisposition disorders that exhibit radiosensitivity with overlapping clinical features including neurological deficits, cellular immunodeficiency with reduction or loss of T- and B-cells, hypogammaglobulinemia, and lymphoid cancers.

### Ataxia-Telangiectasia (AT)

AT is an autosomal recessive disorder with an incidence of 1 per 40,000–100,000 births worldwide, initially described in 1941 by Louis-Bar but coined by Boer and Sedgwick in 1957 (86, 87). AT is a multisystemic disease characterized by ataxia secondary to cerebellar degeneration, telangiectasias, immunodeficiency with recurrent pulmonary infections, premature aging, ionizing radiation sensitivity, and a high risk of developing cancers of lymphoid origin (88). AT is a result of biallelic mutations of *Ataxia Telangiectasia Mutated* (*ATM*) (89), a PI3K-related serine/threonine protein kinase located on chromosome 11q22.3 (90), with a chief function to maintain genomic integrity. Following damage by ionizing radiation, chemotherapy, and oxidative stress (91), DSBs are recognized by MRN complex (MRE11-RAD50-NBS1), which activates ATM (92). Activated ATM amplifies DNA damage signaling by phosphorylating several downstream effectors including cell cycle proteins (Chk1, Chk2) (93), DNA repair proteins (BRCA1) (94), apoptosis (TP53) pathway, and other collaborative DNA damage nodes, including DNA-dependent protein kinase and ATM-related (ATR) (95, 96). Most ATM mutations are truncating and associated with severe or classic phenotype of AT due to a lack of functional kinase. Missense and in-frame mutations allow for some residual ATM activity and are associated with milder clinical course and slow progression (97, 98).

AT classically presents in early childhood, between 1 and 4 years of age, with ataxia manifesting as abnormal gait pattern in a child with otherwise previously normal development. Common neurological symptoms include dysarthria, impaired oculomotor coordination, loss of fine motor skills, and development of sensory and motor neuropathy along with extrapyramidal symptoms. Most patients become wheelchair-bound by the second decade of life (99–102). Telangiectasias are the second most common feature with average onset at 5–8 years of life and occur generally within the bulbar conjunctiva but can also appear on sun-exposed areas such as face and ears (103). Ocular telangiectasias should be differentiated from physiologic ocular vessels due to their constant presence without changing with environment or time. Immunodeficiency is another pronounced feature in two thirds of AT patients, which is demonstrated by a lack of antibody response to vaccines, reduced B and T cell numbers, and decreased production of at least one immunoglobulin subclass (IgG, IgA, and IgM) (104–106). Of note, a minority of AT patients have elevated IgM concurrently with IgA or IgG deficiency, so care must be taken to not misdiagnose these patients as hyper-IgM syndrome (107). Sinopulmonary infections and increased risk of autoimmune or inflammatory diseases, such as ITP, cutaneous granulomatous disease, and vitiligo, is a direct result of immunodeficiency and immune dysregulation (106, 108, 109). Endocrine abnormalities including poor growth, gonadal atrophy, delayed pubertal development, and insulin-resistant diabetes are also common (110–112).

AT patients have a 25% lifetime risk of developing a malignancy, which is the main cause of death in the second or third decade of life along with respiratory insufficiency (113–115). The vast majority of these cancers are of lymphoid origin with B-cell NHL, Hodgkin lymphoma (HL), and ALL occurring at a higher rate in AT patients <20 years of age (113, 114). Strikingly, EBV infection was found to be associated with all HL and half of NHL cases. Other carcinomas including brain, gastric, and liver cancers have been reported (113, 114). Although previously debated, breast cancer is now considered as part of the cancer spectrum with a 30-fold increased risk in AT patients (113). It has been postulated that cancer risk correlates with gene dosage, where patients with classic AT and lack of ATM kinase function are at higher risk of developing lymphoid tumors than patients with some residual AT activity (113).

### Nijmegen Breakage Syndrome (NBS)

NBS is an autosomal recessive disease caused by biallelic mutations in *NBN* located at 8q21.3. *NBN* gene codes for nibrin, which is one of three proteins that make up the MRN complex to activate and recruit ATM to DSBs (116). NBS was named after the Dutch city, Nijmegen, where it was first described in 1981 by Wermaes et al. (117). The prevalence is estimated to be 1 in 100,000 worldwide except in Central and Eastern European Slavic populations where it is more common due to founder mutation with a large cohort in Poland (118, 119).

Microcephaly at birth with distinct, “bird-like” craniofacial features as well as growth retardation and intellectual disability are early features of NBS (120, 121). Immunodeficiency is characterized by severe hypogammaglobulinemia in 20%, IgA deficiency in 50%, and reduced B and T cells in >80% of NBS patients, resulting in a spectrum from silent phenotype to recurrent, chronic respiratory tract infections requiring immunoglobulin replacement (122–124). Malignancy is a significant cause of mortality in NBS patients. More than 40% of patients develop cancer, predominantly of lymphoid origin, by 20 years of age (125). Diffuse large B-cell lymphoma and T cell lymphoblastic lymphoma predominate (126). Other hematological malignancies including HL, B- and T-cell ALL, and AML have also been described (125). Solid malignancies such as medulloblastoma, rhabdomyosarcoma, papillary thyroid carcinoma, glioma, meningioma, neuroblastoma, and Ewing sarcoma occur rarely (125, 127, 128).

### DNA Ligase IV Deficiency (LIGIV)

LIGIV was clinically described in 1990 by Dr. Plowman et al., and in 1999, it was attributed to pathogenic mutations in DNA ligase IV (*LIG4*), located on 13q33.3 (129, 130). LIG4 mediates the final ligation step in the NHEJ pathway, a process utilized not only for NHEJ-mediated DSB repair but also for V(D)J recombination (131, 132). Approximately 40 cases have been reported with hypomorphic LIG4 mutations that correlate with clinical severity (133, 134). Patients present at variable ages with common features including microcephaly, facial dysmorphism, growth failure, infections, and severe immunodeficiency as well as hematological manifestations such as BMF and leukemia/lymphoma (134, 135). The immunologic phenotype can range from a radiosensitive T-B-NK+ severe combined immunodeficiency (SCID) to mild hypogammaglobulinemia and lymphopenia with restricted receptor repertoire (136). Hematological manifestations are largely due to accumulation of ionizing radiation and other genotoxic insults, resulting in BMF in 44% (134, 137, 138) and cancers in 24% of the patient population (134). Cancers of the hematopoietic system are most common and include lymphoid leukemia and lymphomas (EBV positive and negative) and AML (130, 134, 135, 139, 140). Recently, in a cohort of patients with BMF/MDS, a novel homozygous mutation in LIG4 (c.2440C>T, p.R814X) was found in a 10-year-old boy presenting with MDS and monosomy 7 (141).

Genomic efforts have recently uncovered additional mutations in NHEJ repair genes, *Artemis* (*DNA Cross-Link Repair 1C*) and *Cernunnos* (*XLF/NHEJ1*), to cause hematological malignancies in anectodal reports. Compound heterozygous mutations in Artemis (*EX1_3del* and 1384_1390del), a key player in V(D)J recombinase machinery, was shown to cause EBV-associated B-cell lymphoma in a 9-month-old and a 5-year-old patient (142). In a targeted mutation screen in children with hematological cytopenias, a novel homozygous *NHEJ1* mutation (c.236T>C, p.L79P), involved in the final stage of DSB NHEJ repair, was identified as the causative genetic defect in a 21-year-old with MDS and monosomy 7 (143).

## Syndromes Caused by RecQ Helicase Family Deficiencies

Helicases allow access to the genome during replication, recombination, transcription, and repair by unraveling the double helix and other complex DNA and RNA structures in an ATP-dependent manner. RecQ helicases all possess three highly conserved domains: N-terminal ATPase-dependent helicase domain, RecQ-C middle domain with ability to bind various DNA structures, and a C-terminal helicase-and-ribonuclease-D-like (HRDC) domain, which promotes DNA binding stability. *BLM, WRN, RECQL1, RECQL4*, and *RECQL5* are five human RecQ helicases that are essential in maintaining genomic stability during DNA damage repair (144). So far, disease-causing mutations have been described in BLM, WRN, and RECQL4 to cause cancer predisposition syndromes: Bloom, Werner, and Rothmund-Thompson syndrome, respectively.

### Bloom Syndrome (BS)

BS, initially described by Dr. David Bloom in 1954 (145), is an autosomal recessive disorder caused by biallelic mutations in *BLM* located at 15q26.1 (146). As of 2018, almost 300 cases were known to the Bloom Syndrome Registry (147) with predominance of individuals of Eastern European descent, particularly within the Ashkenazi Jewish population who have an estimated carrier rate of 1 in 100 (148). BLM prevents erroneous HR during replication and resolves intermediate DNA structures such as displacement loops and double Holliday junctions (149). In the absence of BLM, dysfunctional HR results in a 10-fold increase in the rate of sister chromatid exchanges (SCEs) compared to healthy individuals (146).

Clinical features of BS include growth failure, sun-sensitive skin rash, endocrine disturbances, and immunodeficiency (150). BS neonates are small for gestational age with normal appearance with some exhibiting feeding difficulties resulting in failure to thrive (148). Photosensitive cutaneous rashes are among the most common manifestations that appear in infancy or early childhood and include telangiectasia erythema of the face (butterfly rash), hands, and forearms, as well as café-au-lait spots and hypopigmented macules (147). Immunodeficiency clinically manifests as frequent upper respiratory and gastrointestinal infections due to dysregulated T cells and hypogammaglobulinemia (particularly IgA and IgM deficiency) (150). Severe chronic lung disease is a common complication of BS thought to be secondary to repeated respiratory infections as a consequence of immunodeficiency (148). In addition to short stature, insulin resistance, type 2 diabetes, dyslipidemia, hypothyroidism, and impaired fertility are well-known endocrine sequalae that develop with age in BS patients (151, 152). Neurologically, BS patients have normal intelligence with very few cases reported with mild intellectual disability (152).

The distribution of cancers in BS patients is similar to that of the general population but with a younger age onset with at least one third of BS patients developing a malignancy by the age of 25 and 80% by the age of 40 years (147). Among 144 BS patients, 223 cancers were reported (147). Hematological cancers were most prevalent, with AML and ALL occurring most frequently with a median age of 18 years followed closely by lymphomas, (predominantly B-cell NHL) with a median age of diagnosis of 20 years (147). Colorectal carcinomas were the next most common solid tumors found in 28 of 223 cancers, with a median onset age of 37 years. Other common neoplasms include breast cancer, non-melanomatous basal and squamous cell skin carcinomas, and Wilms tumor (147).

### Werner Syndrome (WS)

WS, previously known as adult onset progeria with cancer predisposition, is an autosomal recessive disorder initially reported by German medical student Otto Werner in 1904. He described a family of four siblings in their third decade of life that exhibited signs of premature aging, with graying of the hair, bilateral cataracts, scleroderma, and short stature (153), which was later attributed to biallelic mutations in the Werner (WRN) helicase (154). The prevalence is estimated at 1:380,000–1:1,000,000 (155) and is higher in the Japanese (156) and Sardinian (157) population with an estimated frequency of 1:20,000–1:40,000 and 1:50,000, respectively. More than 70 different pathogenic mutations were found in the helicase and exonuclease domains of *WRN* located on locus 8p12 (158, 159). WRN has well-established functions in several DNA repair pathways, including NHEJ (158), HR (160), BER (161), and telomere maintenance (162).

The first presenting sign of WS is often short stature in a pre-adolescent individual failing to undergo a growth spurt. By the early third decade, ectodermal changes will become prominent featuring skin atrophy, graying or loss of hair, and bilateral cataracts (154) with readily discernable bird-like facies. Skin atrophy and calluses, which can progress to intractable ulcers, are common along with Achilles tendon calcification, a highly characteristic of WS in older patients (163). Common older age-associated endocrine abnormalities appear in the late 30s, including type II diabetes, osteoporosis, and hypogonadism causing infertility (154, 163). Furthermore, WS patients suffer from premature and severe forms of atherosclerosis and medial artery calcification (154, 164). Surprisingly, there is a paucity of neurodegenerative changes in these patients in addition to lack of skeletal anomalies or intellectual disability (154, 165). Heart attacks and malignancies are the leading cause of morbidity in WS patients resulting in a low median life expectancy of 54 years (164). WS patients have a 2–60-fold increased risk for neoplasms, with thyroid follicular carcinomas as the most common cancer followed by melanoma, meningioma, sarcomas, leukemia/MDS, and primary bone tumors (166, 167).

The International Registry of Werner Syndrome has provided five cardinal signs for WS diagnosis in individuals >10 years of age: bilateral cataracts, characteristic skin changes, short stature, parental consanguinity or affected siblings, and premature hair graying (154). More than 90% of affected individuals had four cardinal features (154, 164). There is a subgroup of patients classified as atypical Werner syndrome (AWS), which is used to describe individuals with a clinical diagnosis of WS but a lack an identifiable *WRN* mutation. Of the 71 patients with AWS, a subset was shown to carry mutations in *LMNA*, a gene known to be mutated in the Hutchinson-Gilford Progeria syndrome (HGPS) (168), or in *POLD1*, a DNA polymerase involved in several DNA repair pathways (169). Thus, far, malignancies have not been reported among these AWS patients (154).

### Rothmund Thompson Syndrome (RTS)

RTS was initially described by the German ophthalmologist Dr. August von Rothmund in 1868 with unique ectodermal features followed by a similar description by Dr. Sydney Thomson, British dermatologist, in 1921. It was not until 1957 when Dr. Taylor coined the syndrome, which now has almost 500 patients described in all ethnicity groups (170). RTS results from autosomal recessive germline mutations in *RECQL4*, which organizes the DNA replication machinery, promotes DNA end resection with MRN and CtIP complex during HR and promotes NHEJ in G1 phase of the cell cycle (171, 172).

Cutaneous rash is the hallmark clinical sign in RTS, which commonly presents in infancy with an erythematous facial rash that spreads to buttocks and extremities while sparing the trunk. The rash progresses to poikiloderma (reticulated hypo- and hyperpigmentation, telangiectasias, and punctate atrophy) over months to years and persists throughout life. Hyperkeratotic lesions and café-au-lait spots can manifest later (170, 173). Skeletal abnormalities and long bone defects were found in 75% of RTS patients (174). Ocular abnormalities occur with varying prevalence of 10–50% with rapid-onset bilateral cataracts being most frequent (175). Other common features include short stature, sparse or absent hair, dental anomalies, and feeding difficulties (176, 177). Immunodeficiency is uncommon, although IgG and IgA deficiencies along with T-B+NK-combined immunodeficiency have been described (178–180). The most common malignancy among RTS patients is osteosarcoma with a prevalence of 30%, occurring at a younger median age of 11 years compared to the general population (177). Skin cancers, including melanoma and basal cell and squamous cell carcinoma, constitute the second most common cancer affecting 5% of patients (177, 181, 182). Rare hematological malignancies include MDS, lymphomas (NHL, HL), and AML (173).

Notably, germline mutations in *RECQL4* gene had also been associated with two other constitutional disorders with lymphoma risk. First, RAPADILINO (**RA**dial **RA**y defect; **PA**tellae hypoplasia or aplasia and cleft or highly arched **PA**late; **DI**arrhea and **DI**slocated joints; **LI**ttle size and **LI**mb malformation; **NO**se slender and **NO**rmal intelligence) syndrome. It has been initially described in Finland in 1989 (183) to affect an estimated 1 in 75,000 individuals and manifest with pre- and post-natal growth failure, cervical spine defects, failure to thrive, and juvenile diarrhea of unknown cause (184). Lymphoma was reported in 4 patients and osteosarcoma in 1 patient with RAPADILINO syndrome (185). Second, Baller–Gerold syndrome (BGS), first reported by Cohen in 1975, was based on three patients described in 1950 by Baller and 1959 by Gerold in German literature (186). Fewer than 40 patients have been described with an unknown prevalence (187). BGS patients with RECQL4 mutations have craniosynostosis, upper-limb anomalies, short stature, and poikiloderma (188). Thus, far, only one case of malignancy (NK/T-cell lymphoma) has been reported in a 2.5-year-old individual with BGS (189).

## Cancer Risk Among Heterozygous Mutation Carriers

Individuals with germline heterozygous (monoallelic) mutations in some DNA repair genes have an increased lifetime risk of cancer, which is often facilitated by the acquisition of a somatic mutation affecting the remaining wild-type allele. The spectrum and onset age of cancers in individuals with heterozygous mutations differ compared to individuals with biallelic mutations in the same gene. Genetic counseling is recommended for all patients with, or at risk for having, monoallelic or biallelic DNA repair disorders due to the complex nature of these conditions and their associated health risks (190).

Cancer screening guidelines have been established by multiple organizations to address the need for increased surveillance and/or prophylactic management for these high-risk individuals (191–193). Gene-specific cancer screening guidelines have also been established internationally for individuals with monoallelic variant for a DNA repair disorder gene with high risk of cancer development (194–196). Many of these guidelines are region specific and may differ from recommendations, when available, in other parts of the world. Continued efforts to harmonize these recommendations are needed to ensure patients have access to appropriate management worldwide.

Monoallelic pathogenic mutations in the mismatch repair genes, *MLH1, MSH2, MSH6, PMS2*, and *EPCAM*, are associated with Lynch syndrome, a cancer predisposition syndrome characterized by an increased risk of colon cancer, uterine cancer, ovarian cancer, genitourinary tract cancers, and other gastrointestinal cancers. Cancer risk varies among the different MMR genes. Heterozygous mutations in PMS2, for instance, are associated with a lower risk of colon and endometrial cancers and are often diagnosed at later ages than in individuals with heterozygous mutations in MLH1 or MSH2 (197, 198).

Heterozygous mutations in FA genes involved in DSB repair predispose to development of breast, ovarian, and other cancers. These include *BRCA1* and *BRCA2* mutations that confer a 50–80% lifetime breast cancer risk, 10–40% lifetime ovarian cancer risk, and increased risk of male breast, pancreatic, and prostate cancer, as well as melanoma (199, 200). Heterozygous loss of *PALB2* has also been demonstrated to confer a susceptibility to breast and pancreatic cancer, as *PALB2* interacts directly with both *BRCA1* and *BRCA2* during HR. An elevated risk of later onset serous ovarian cancer has been demonstrated in individuals with heterozygous loss-of-function *BRIP1* mutations (201). Biallelic mutations in *BRCA1, BRCA2, PALB2*, and *BRIP1* result in FA groups S, D1, N, and J, respectively. Recent meta-analyses have estimated that the lifetime risk of breast cancer in *ATM* heterozygotes is 33–38% (115), although the c.7271T>G mutation may be associated with a significantly higher breast cancer risk (202). Heterozygous *ATM* mutations may also confer a susceptibility to pancreatic cancer (203). Heterozygous carriers of the *NBN* c.657del5 mutation (which is found in homozygous state in more than 90% of patients with Nijmegen breakage syndrome) who also carry two copies of the NBN polymorphism p.E185Q (GG allele) were shown to be at increased risk for breast and prostate cancers (204, 205). These recent studies are the first clear example of genetic modifier effect in a germline cancer syndrome, where the penetrance of a heterozygous allele is “activated” by the presence of an additional modifying polymorphism in the same gene.

## Diagnostic Considerations

### History and Examination

A thorough patient history, family history, and physical examination gives the first suspicion or a “red flag” pointing to an underlying DNA repair disorder (Table 2). Multisystem history should be obtained along with birth and developmental history since manifestations can appear at any location during the lifetime. If the patient has been treated for prior malignancy, age of diagnosis, type and location of cancer, treatment history, and hypersensitivity to chemotherapeutic agents should also be addressed. Family history features suggestive of one of these conditions include the presence of early-onset cancers in family members, multiple family members with cancer, or multiple cancers in one individual. Other concerning features include the presence of immunodeficiency, neurologic abnormalities, or deaths in young children from medical or unknown causes. Familial consanguinity should be noted because many of the DNA repair disorders are inherited in an autosomal recessive manner. Consideration should be given to the family's ethnic background as some of these disorders are enriched in specific ethnic populations secondary to founder mutations. Physical exam findings concerning DNA repair disorder include facial dysmorphology (particularly microcephaly, which should be evaluated by measuring head circumference); absent, sparse, brittle, or prematurely gray hair; as well as numerous dermatologic findings such as café-au-lait macules, hypopigmentation, multiple lentigines, telangiectasias, or rashes, especially if occurring on the face. An accurate height should also be obtained, as many patients with a DNA repair disorder are of short stature. Suggestive neurologic findings include loss of deep tendon reflexes, spasticity, ataxia, or other gait changes. Referral to a clinical geneticist may also be of benefit to further assess for features of these conditions.

**Table 2:** The presence of multiple red flags in the medical and/or family history increases concern for an underlying DNA repair disorder and should warrant further evaluation.

	**“Red flags”**
Constitutional features	Short stature Microcephaly Sparse or premature gray hair
Skin	Photosensitivity Pigmentation changes (hypo/hyperpigmentation) Poikiloderma Café-au-lait spots Teleangiectasias Pilomatricoma/pilomatrixoma (benign, hair follicle associated tumor) Butterfly shaped facial skin rash
Neurologic	Intellectual disabilities Hyporeflexia Loss of fine or gross motor skills Ataxia
Immunodeficiency	Recurrent sinopulmonary infections Hypogammaglobulinemia T and B lymphocytopenia
Hematologic	Bone marrow failure
Cancers	Pediatric cancers including head and neck, brain, squamous cell carcinoma, melanoma, adrenocortical carcinoma, NHL, MDS, AML Family member with cancer below age 50, especially if of breast, endometrial, or colorectal origin 2 or more cancers in one individual/family Multiple family members with similar or related cancers

### Functional Assays

Functional testing aids in the diagnostic workup of DNA repair disorders (Table 1). Telomere length is an important diagnostic tool that is used to diagnose short telomere syndromes such as dyskeratosis congenita, a BMF syndrome with mucocutaneous fragility and symptoms of premature aging with an increased predisposition to malignancies secondary to genetic deficiencies in telomere-associated genes such as *TERT, TERC, DKC1, TINF1*, and *RTEL1* to name a few [excellent review provided by (206)]. Importantly, telomere length should be measured in DNA repair disorders such as FA (207, 208), AT (209, 210), NBS (211), BS (212), and WS (213) where patients exhibit short telomeres and chromosome end fusions secondary to dysfunctional DNA damage response at the telomere.

Chromosome breakage studies are necessary to establish a diagnosis of FA, as individuals with this condition are hypersensitive to crosslinking agents such as mitomycin C (MMC) or diepoxybutane (DEB). When exposed to these agents, patient cells will have an increased rate of chromosome breaks and aberrations such as radial figures and rearrangements. Rarely, mosaicism can occur in lymphocytes where two distinct lymphocyte populations are present with one subset having undergone spontaneous reversion resulting in normal sensitivity to clastogenic agents while the second population remains with the underlying genetic defect and retaining hypersensitivity features to damaging agents. Therefore, if breakage studies on lymphocytes are normal but there is still clinical suspicion for a DNA repair disorder, skin fibroblasts should be investigated to complete the diagnostic evaluation (76).

DNA repair disorders that present with profound immunodeficiency [AT, NBS, NHEJ deficiencies (Ligase IV, Artemis, Cernunnos)] can lead to absence or very low T-lymphocyte receptor excision circles (TRECs), which are detected on newborn screen (214, 215).

Spontaneous excess of immunoglobulin (Ig)/T-cell receptor (TCR) abnormal rearrangements of chromosomes 7 and 14 are common in patients with NBS (10–35%) (216) and AT (5–10%) (217). Alpha fetoprotein (AFP) is elevated in 95% of AT patients (218), but interestingly, it can also be increased in FA patients (219). Sister chromatid exchange (SCE) assay, which assess for increased SCE in metaphase cells with bromodeoxyuridine (BrdU) exposure, aids in the diagnosis of BS (148). UV hypersensitivity assay, where skin fibroblasts are exposed to UV light, is used for diagnosing NER defect in XP patients, but this testing is typically completed in a research setting and may not be available clinically (220). There is a lack of consensus and uniform availability for a routine radiosensitivity assay available for patients with HR and NHEJ biallelic genetic disorders. Radiation-induced lymphocyte apoptosis (RILA) assay and phospho-ATM assay have some predictive potential (221). Analysis of radiation-induced γH2AX foci accumulation in T and NK lymphocytes of LIG4-SCID individuals was recently implemented as a flow cytometry assay (222).

### Genetic Testing

It has become a standard approach to perform genetic studies as part of the initial diagnostic workup in a patient with a suspected DNA repair disorder based on clinical features and/or history of related malignancies. The patient's clinical phenotype and results of functional testing can be used to guide the differential diagnosis and, in turn, the genes requiring further investigation. Genetic testing of individuals presenting with a related malignancy but lacking other clinical manifestations of a DNA repair disorder is unlikely to have a high yield, as these conditions are thought to be rare. However, the diagnostic pickup of a DNA repair disorder in individuals with a related malignancy in an unbiased manner requires further study.

When ordering genetic testing, issues to consider include sample source, optimal genetic testing type, and technical challenges limiting mutation identification. First, peripheral blood or saliva samples are the easiest and most preferred sample source to obtain. In patients with active hematologic malignancy, however, skin fibroblasts or hair follicles are the preferred germline specimen (223). Single gene analysis may be an appropriate rapid approach in scenarios where a specific gene is expected based on phenotype. A disease-specific multigene panel is a cost-effective approach for patients with clinical features consistent with multiple DNA repair disorders. Currently, clinical whole exome or genome sequencing represent the most comprehensive approach, generally used after obtaining negative results from targeted gene testing. Some genes may present technical challenges, such as the *PMS2* gene, which has multiple pseudogenes. One of these pseudogenes, *PMS2CL*, is part of a 100-kb inverted duplication and has close sequence homology to the regions of exons 9 and 11–15 in *PMS2*, making it difficult to differentiate whether the mutation is located within *PMS2* or the pseudogene (224).

When interpreting variants obtained in genetic studies, it is widely accepted to use consensus criteria established by the American College of Medical Genetics and Genomics to classify variants as pathogenic, likely pathogenic, variant of uncertain significance (VUS), likely benign, and benign (225). Pathogenic and likely pathogenic variants will confirm a clinical diagnosis and thus impact medical management decisions. If a patient with a suspected autosomal recessive DNA repair disorder is found to have a heterozygous pathogenic mutation in a gene consistent with the phenotype, one has to consider that a second mutation within the same gene was missed. A discussion with the reporting lab may be helpful to clarify limitations of their testing strategy and whether additional testing may be warranted to evaluate for a second gene alteration, which might include not only a mutation but also an intragenic deletion or intronic variant. An increasingly growing challenge in the clinical setting is the finding of a VUS, for which the available genetic and functional data are either lacking or conflicting and, therefore, at a given time, they generally should not influence clinical decision making. However, periodic communication with the testing lab is encouraged to learn of any changes in variant interpretation that may occur over time.

## Treatment Strategies

A unifying feature among most DNA repair disorders is hypersensitivity to DNA-damaging agents such as radiation and chemotherapy used to treat malignancies. However, the underlying genetic deficit of repair pathway genes in patients with DNA repair syndromes places them at high risk for therapy-related toxicities. For this reason, unique cancer treatment regimens are tailored that often employ reduced intensity doses to balance chemo- or radiotherapy-mediated toxicities while achieving clinical outcomes comparable to the standard of care. The high rate of treatment failures and secondary malignancies is problematic, especially in patients with CMMRD, NBS, and AT. Common strategies to avoid overt toxicities include avoiding radiomimetic drugs such as bleomycin and dactinomycin and being aware of cyclophosphamide- and/or ifosfamide-related hemorrhagic cystitis developing outside the normal range in patients with predisposition to telangiectasias. DSB DNA repair syndromes (AT, NBS, and LIGIV), due to their shared manifestations of immunodeficiency and increased risk for malignancies, benefit from reduced intensity conditioning-based hematopoietic stem cell transplantation (HSCT). However, the role of HSCT in improving overall outcome of patients with AT remains debatable (215). Several clinical trials are aimed at innovative drugs that target DNA repair genes to provide effective therapy while minimizing toxicities for patients with DNA repair disorder-associated cancers (226).

## Conclusions

Cancer can result from mutations that are inherited or acquired during lifetime. DNA repair mechanisms are essential to maintenance of genomic integrity and are abrogated in cancer. Defects in DNA repair pathways result in a chaotic and unstable genomic environment, which is a hot bed for oncogenic transformation. This biological phenomenon is well-recapitulated in classic DNA repair disorders that result from heritable mutations in genes essential for DNA damage response and result in early-onset cancers and premature aging. Because these syndromes are rare, a heightened awareness must be practiced to provide multidisciplinary care and surveillance and unique therapeutic considerations for patients with DNA repair disorders.

## Author Contributions

RS and SL wrote and revised the manuscript. All authors contributed to the article and approved the submitted version.

## Conflict of Interest

The authors declare that the research was conducted in the absence of any commercial or financial relationships that could be construed as a potential conflict of interest.
